# The radioresistance kinase TLK1B protects the cells by promoting repair of double strand breaks

**DOI:** 10.1186/1471-2199-6-19

**Published:** 2005-09-12

**Authors:** Gulshan Sunavala-Dossabhoy, Sri Kripa Balakrishnan, Siddhartha Sen, Sam Nuthalapaty, Arrigo De Benedetti

**Affiliations:** 1 Department of Biochemistry and Molecular Biology and the Feist-Weiller Cancer Center, Louisiana State University Health Sciences Center. 1501 Kings Highway, Shreveport, LA 71130-3932, USA

## Abstract

**Background:**

The mammalian protein kinase TLK1 is a homologue of *Tousled*, a gene involved in flower development in *Arabidopsis thaliana*. The function of TLK1 is not well known, although knockout of the gene in *Drosophila* or expression of a dominant negative mutant in mouse cells causes loss of nuclear divisions and missegregation of chromosomes probably, due to alterations in chromatin remodeling capacity. Overexpression of TLK1B, a spliced variant of the TLK1 mRNA, in a model mouse cell line increases it's resistance to ionizing radiation (IR) or the radiomimetic drug doxorubicin, also likely due to changes in chromatin remodeling. TLK1B is translationally regulated by the availability of the translation factor eIF4E, and its synthesis is activated by IR. The reason for this mechanism of regulation is likely to provide a rapid means of promoting repair of DSBs. TLK1B specifically phosphorylates histone H3 and Asf1, likely resulting in changes in chromatin structure, particularly at double strand breaks (DSB) sites.

**Results:**

In this work, we provide several lines of evidence that TLK1B protects the cells from IR by facilitating the repair of DSBs. First, the pattern of phosphorylation and dephosphorylation of H2AX and H3 indicated that cells overexpressing TLK1B return to pre-IR steady state much more rapidly than controls. Second, the repair of episomes damaged with DSBs was much more rapid in cells overexpressing TLK1B. This was also true for repair of genomic damage. Lastly, we demonstrate with an *in vitro* repair system that the addition of recombinant TLK1B promotes repair of a linearized plasmid incubated with nuclear extract. In addition, TLK1B in this *in vitro *system promotes the assembly of chromatin as shown by the formation of more highly supercoiled topomers of the plasmid.

**Conclusion:**

In this work, we provide evidence that TLK1B promotes the repair of DSBs, likely as a consequence of a change in chromatin remodeling capacity that must precede the assembly of repair complexes at the sites of damage.

## Background

The *Tousled *gene of *Arabidopsis thaliana *encodes a protein kinase which, when mutated, results in abnormal flower development characterized by a stochastic loss of floral meristem and organs [1]. Two mammalian *Tousled*-like kinases (TLK1 and TLK2) were cloned by Sillje *et al*., 1999 [2] during a PCR-based search for human kinases, who also reported that the activity of these kinases is maximal in S phase, and more recently, these kinases were reported to be targets of checkpoint kinases, ATM and Chk1 [3]. Specifically, it was reported that TLK1 is inhibited by Chk1 by direct phosphorylation at S^695^. These findings identify a functional cooperation between ATM and Chk1 in propagation of a checkpoint response to DNA damage and suggest that through transient inhibition of TLK1 the ATM-CHK1-TLK pathway may regulate processes involved in chromatin assembly [4]. Indeed, in AT cells (cells deficient in ATM protein) TLK1 was not inhibited after genotoxic stress [4]. Since ATM and Chk1 are involved in the DNA damage checkpoint upon radiation, this suggests that TLKs may be involved in some aspect of genome surveillance, particularly chromatin remodeling concurrent with DNA repair (see below). The function of TLK1 is not well known, although knockout of the gene in *Drosophila *or expression of a dominant negative mutant in mouse cells causes loss of nuclear divisions and missegregation of chromosomes [5,6] likely through changes in chromatin remodeling capacity. The importance of TLK1 in chromosome segregation was recently confirmed in *C. elegans *embryos [7].

We recently cloned a cDNA encoding a mammalian *Tousled*-like kinase (TLK1B) through a very different scheme than the one used by Sillje *et al*. [2], based upon polysomal redistribution of weakly translated transcripts that become preferentially recruited upon overexpression of eIF4E [8]. Indeed, the human TLK1B mRNA (a splice variant of the TLK1 mRNA cloned by Sillje *et al*.) contains a 5'UTR 1088-nt-long with two upstream AUG codons, which was found to be very inhibitory for translation [8,9]. The inhibition of translation could be relieved by either overexpressing eIF4E, or by deleting a large section of the 5'UTR [8]. We subsequently discovered that TLK1B overexpression protects the cells from the genotoxic effects of ionizing radiation (IR) or the radiomimetic drug, doxorubicin. TLK1B probably exerts these effects by phosphorylating histone H3 [8,10] and the histone H3 chaperone Asf1 [10,11], and thereby promoting chromatin remodeling concurrent with repair of DNA damage. Interestingly, synthesis of TLK1B is induced at the translation level by the presence of double strand breaks [DSBs; [12]].

The discovery that TLK1B is a kinase that phosphorylates histone H3 came first in a series of experiments aimed at identifying the potential substrates of TLK1B. We carried out kinase assays with recombinant GST-TLK1B and various typical substrates, only a few of which were phosphorylated efficiently. However, we found that TLK1B phosphorylated very well histone H3 at S^10 ^but not the other core histones or H1 [8]. We subsequently confirmed that the MM3MG cells overexpressing TLK1B had a higher constitutive level of H3 phosphorylation in asynchronous cells [8]. We could also show genetically that TLK1B is a histone H3 kinase. We showed that inducible expression of TLK1B in a yeast strain carrying a temperature-sensitive allele of the major H3 kinase [Ipl-1; [13]] could rescue growth at the non-permissive temperature [8]. In addition, it restored normal levels of histone H3 phosphorylation [8]. Significantly, expression of TLK1B in yeast increased radioresistance, indicating a conservation of function and substrates. Interestingly, we also found that IR results in a loss of H3 phosphorylation, but the significance of this result was unclear. The dephosphorylation of H3 after IR was reported also by another group [14]. Recently, a possible explanation for this effect was described [3,4]. These authors showed that TLK1 (the larger isoform) is inhibited by γ-radiation. The inhibition is presumably mediated by ATM and Chk1 by direct phosphorylation at S^695 ^[4]. It seemed possible that physiologically the increased TLK1B synthesis following IR can help offset the loss of TLK1 activity resulting from IR and restore appropriate levels of histone H3 phosphorylation.

In this paper we present evidence that overexpression of TLK1B protects the cells from IR by facilitating the repair of DSBs, and that the steady state phosphorylation of H3 and H2AX is restored to pre-IR much more rapidly in TLK1B overexpressing cells.

## Results

### TLK1B protects cells from IR

**Figure 1:**
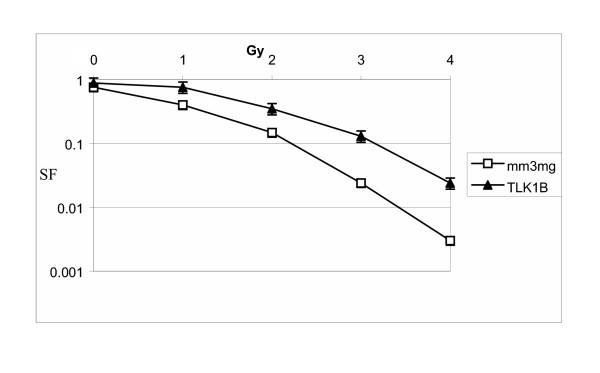
**Pattern of γ-radiation sensitivity by clonogenic assays**. 10^4 ^untransfected MM3MG and cells overexpressing TLK1B were irradiated with the indicated doses and plated in triplicates of 100, 500, 1000, or 5000 cells in multiwell plates. The colonies were counted 10 days later. The average from 2 independent experiments is shown. S.F. = surviving fraction.

We previously published initial evidence that TLK1B protects a normal mammary cell line (MM3MG) from IR [8]. This result is now reproduced in Fig. 1 in a more complete assay with serially diluted cells plated for clonogenic assays. We show that 90% of untransfected MM3MG were killed at 2 Gray (Gy), in contrast to only 55% of those transfected with TLK1B (P = 0.001 by one-tailed t-test). At 4 Gy, very few MM3MG cells survived, in contrast to 4% of cells expressing TLK1B (P = 0.0001). This confirmed that overexpression of TLK1B significantly increased their radioresistance.

### Inverse phosphorylation of H2AX and H3 after IR

**Figure 2:**
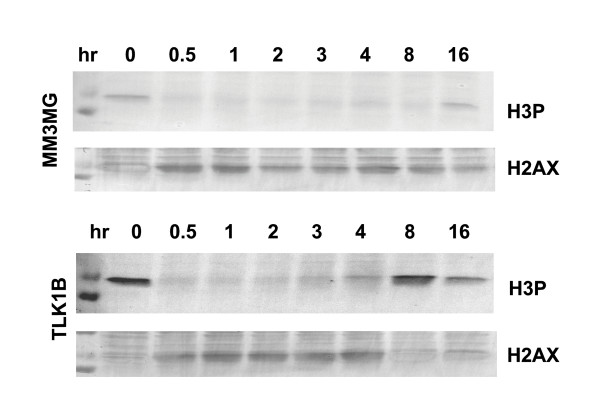
**Phosphorylation of H3 and H2AX after irradiation**. MM3MG and MM3MG over-expressing TLK1B cells were grown to 80% confluence prior to gamma-radiation (10 Gy). Cells were harvested at different times (0 h, 30 min, 1 h, 2 h, 4 h, 8 h and 16 h) after irradiation and lysed in RIPA buffer. Equal amount of protein of each sample was loaded on a 15% SDS-PAGE gel and electrophoresed. Blots were probed with phospho-Histone H3 (Ser-10) antibody, or phospho-Histone H2AX (Ser-139) antibody (Upstate Cell Signaling). Equal loading of proteins was confirmed by staining the blots with Ponceau S prior to processing. These blots are representative of two separate experiments.

We previously reported that TLK1B phosphorylates very well histone H3 at S^10 ^but not the other core histones or H1 [8]. Interestingly, we also found that IR results in a loss of H3 phosphorylation, but the significance of this result was unclear. If TLK1 is the major kinase involved in a "chromosomal response" to DNA damage, then inhibition of TLK1 or TLK1B activity by IR through ATM is expected to result in a loss of phosphorylation of histone H3. To probe for the significance of the H3 dephosphorylation, we carried out a time course of recovery from IR and monitored the phosphorylation of H2AX and H3 in the MM3MG cells that overexpress TLK1B. Radiation-induced damage has been shown to result in rapid phosphorylation of histone H2AX at DSB sites [15,16], while BRCA2, a protein that localizes to DSBs, was found to accumulate to sites of condensed chromatin colocalized with phosphorylated H3 [17]. As seen in Fig. 2, phosphorylation of H2AX is, as expected, very rapid after IR in both control and TLK1B cells. In the control, phosphorylation of H2AX remains elevated for at least 8 hr and slightly reduced after 16 hr of recovery. Further, phosphorylation of H3 drops drastically after IR and only recovers after 16 hr in the control. In TLK1B cells, basal phosphorylation of H3 is elevated at t = 0, as previously reported [8] and then decreases after IR (note that the Chk1 phosphorylation site is conserved in TLK1B). However, between 4 hr and 8 hr, the phosphorylation of H3 recovers completely. At the same time, phosphorylation of H2AX drops precipitously. This suggests that repair of DNA is much more rapid in TLK1B cells, if the phosphorylation state of histones H3 and H2AX and presumed remodeling of chromatin can be taken as an indicator of DSB repair.

### Blocking ATM activity with wortmannin prevents the loss of H3 phosphorylation after IR

**Figure 3:**
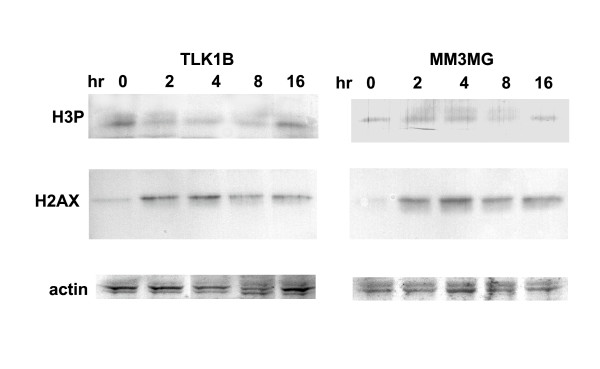
**Phosphorylation of H3 and H2AX after irradiation in the presence of wortmannin**. The experiment was carried out as detailed in the legend to Fig. 2, but the cells were pre-treated with 30 μM wortmannin to inhibit ATM.

If TLK1 and TLK1B are kinases that are largely responsible for the phosphorylation of H3 in unstressed conditions (excluding mitosis), and if activation of ATM following IR is responsible for inhibiting TLK1/B [as previously published; [3,4]], then inhibition of ATM with wortmannin should prevent in large part the dephosphorylation of H3 following IR. Fig. 3 shows that this is indeed the case, since cells pretreated with wortmannin and subjected to IR showed only a very modest decrease in H3 phosphorylation throughout the time course of IR and recovery. Contrary to the expectation, phosphorylation of H2AX was not inhibited by wortmannin in either control or cells overexpressing TLK1B. This was somewhat surprising since ATM is believed to directly phosphorylate this histone after IR [18]. However, very recently, it was demonstrated that ATR, which requires much higher concentrations of wortmannin for inhibition (0.1 mM), also phosphorylates H2AX after radiation, although with somewhat delayed kinetics [16].

### Episomal vectors as reporters for DSBs

To overexpress TLK1B, we used an episomal vector called BK-Shuttle [19]. An important advantage is that these plasmids can be easily rescued from mammalian cells by the Hirt's supernatant protocol [19]. Most stable cell lines carrying these vectors typically have hundreds of copies of the episomes, which have the typical structure of mini-chromosomes and thus behave like genomic DNA [20,21]. We previously showed that the extraction of episomes is a very simple and efficient protocol that yields very consistent amounts of plasmids that are linear with respect to cell numbers [19]

**Figure 4:**
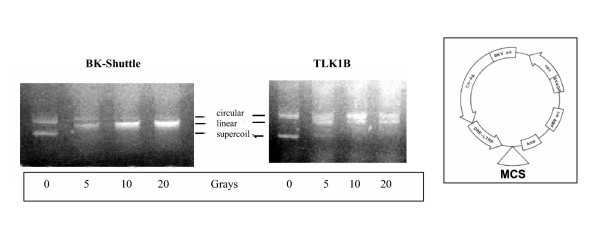
**Analysis of episomes**. 2 × 10^7 ^cells transformed with BK-Shuttle or TLK1B were irradiated with the indicated dose of γ-radiation. The cells were returned to the incubator and the plasmids were isolated 1 hr later by the Hirt's protocol and separated on a 1% agarose/TAE gel. The mobility of the forms (circular, linear, and supercoiled) is indicated. The structure of the BK-Shuttle episomal vector is shown on the right. The bands were quantified with ImageQuant vs. 5 (Molecular Dynamics).

In Fig. 4, we show an example of how these episomes can be used to study protection from IR-mediated DSBs. We have found that IR causes significant damage to the episomes, as shown by a large proportion of linearized molecules due to DSBs. Plasmids isolated from untreated cells migrate as a predominant supercoiled fast-migrating form and a relaxed circular form. Plasmids isolated from irradiated cells show a dose-dependent loss of the supercoiled form due to DSBs, which convert the plasmid to a linearized form (and some nicked circular). The conversion of closed (circular and supercoiled forms) to linearized plasmids can be exploited to monitor the activity of TLK1B. Since the episomes are assembled in typical chromatin, the function of TLK1B in repair (*e.g*., chromatin remodeling to produce ends suitable for ligation) would mirror its function on genomic DNA. Note that the intensities of the bands were quantified with an imaging program (ImageQuant), and that the sum of the bands (supercoiled, linear, or circular) was a constant, indicating very consistent yields of episomes.

### Episomal vectors as reporters for DSB repair

**Figure 5:**
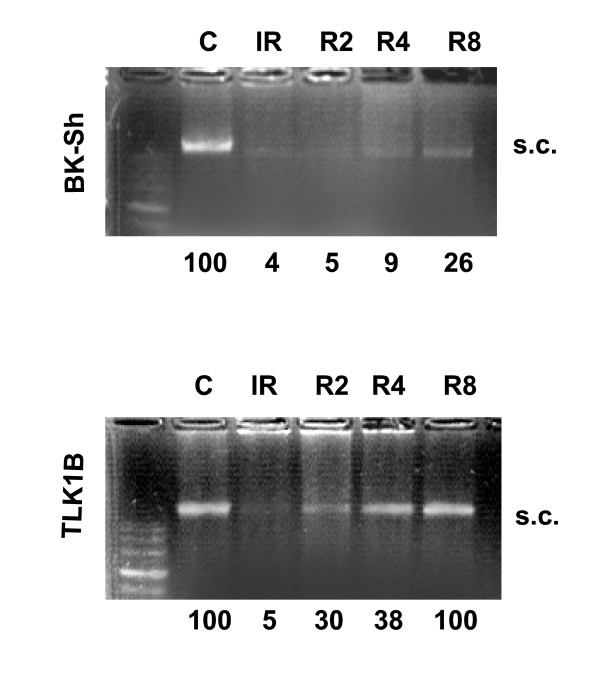
**Analysis of episomes during a time course of recovery from IR**. 2 × 10^7 ^cells transformed with BK-Shuttle or TLK1B were irradiated (or not, **C**) with 20 Gy of γ-radiation. The cells were returned to the incubator and the plasmids were isolated immediately after radiation (**IR**), or after the indicated times of recovery, 2 to 8 hr (**R2-R8**). The episomes were recovered by alkaline lysis, which removes plasmids with DSBs by strand separation followed by the rapid renaturation. This is because it is not always easy to separate on agarose gels the linearized from the supercoiled form of the plasmid (these plasmids are 11–14 kb in size, without and with insert). The bands were quantified with ImageQuant. The intensities of the bands relative to control are indicated underneath each lane. Size markers (Novagen 1 kb ladder) are shown in the first lane.

The episomal vectors can be used as reporters for repair of DSBs and provide a useful system to test if this is the likely mechanism of radioresistance. Cells were irradiated and allowed to recover during an 8 hr time course (repair time), and plasmids were isolated for analysis by gel electrophoresis. In this experiment, however, the episomes were extracted from cells by alkaline lysis (essentially the same protocol used to extract plasmids from bacteria). Plasmids that are linearized with a DSB or plasmids with nicks are not recovered from alkaline lysis because of strand separation. In contrast, supercoiled/covalently closed plasmids are recovered from alkali treatment. Fig. 5 shows that IR causes an immediate and significant loss of supercoiled plasmids in control MM3MG cells containing the empty vector. Religation of the linear plasmids and formation of the supercoiled molecules does not occur until 4 to 8 hr of recovery from IR, and then, the recovery is only partial. In contrast, IR causes a similar loss of supercoiled molecules at t = 0 in MM3MG cells expressing TLK1B, but the recovery is very fast. At 2 hr (R2) the recovery of supercoiled molecules is almost complete and it is fully restored by 8 hr (R8). The recovery of the supercoiled forms is almost certainly due to the repair of pre-existing, damaged plasmids and not due to de novo synthesis. First, the repair time was too short to account for a large fraction of newly synthesized plasmids, and second, IR results in a dramatic arrest in DNA replication in normal cells [22], making it highly unlikely that any newly replicated plasmid was achieved. This experiment was repeated with identical results, indicating that the loss of supercoiled species after IR can be easily detected by alkaline lysis with great reproducibility.

### Enhanced repair of genomic damage in TLK1B cells

**Figure 6:**
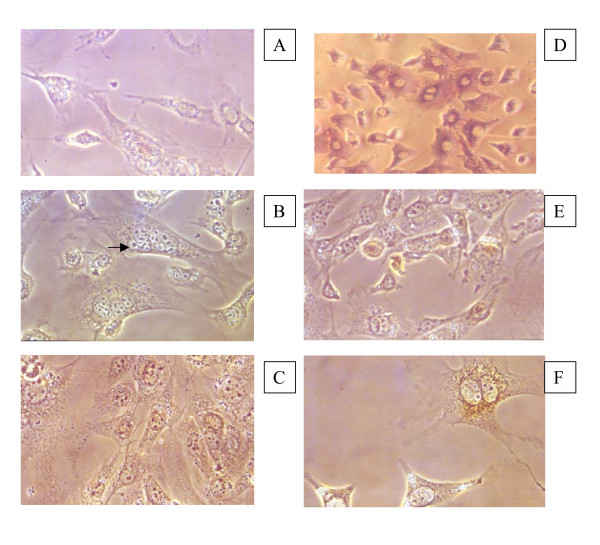
**Assay of repair of genomic damage**. Examples of non-irradiated cells (A, D) or cells irradiated and allowed to recover for 0 hr (B, E) or 2 hr (C, F) are shown. MM3MG cells are shown in A-C; TLK1B cells are shown in D-F. An arrow points to one of the DAB spots.

To assess genomic repair, a modified terminal deoxytransferase (TdT) fill-in reaction was adopted. Briefly, the cells were irradiated (or not) to create genomic breaks, which were then processed with TdT and biotinylated dNTPs according to the manufacturer's protocol (see Methods). Macroscopic deposit of diaminobenzidine (DAB) at breakage sites was used to determine the extent of genomic damage and repair during a time course of recovery from IR (Fig. 6 and Table 1). No DAB deposits were visible in non-irradiated cells. In irradiated cells, 9–13 spots per cell were visible immediately after IR in both control and cells expressing TLK1B. Already at two hr of recovery from IR, in TLK1B cells the number of spots decreased by more than 50%, in contrast to control cells in which there was no appreciable recovery.

### Rapid repair and chromatin assembly *in vitro*

**Figure 7:**
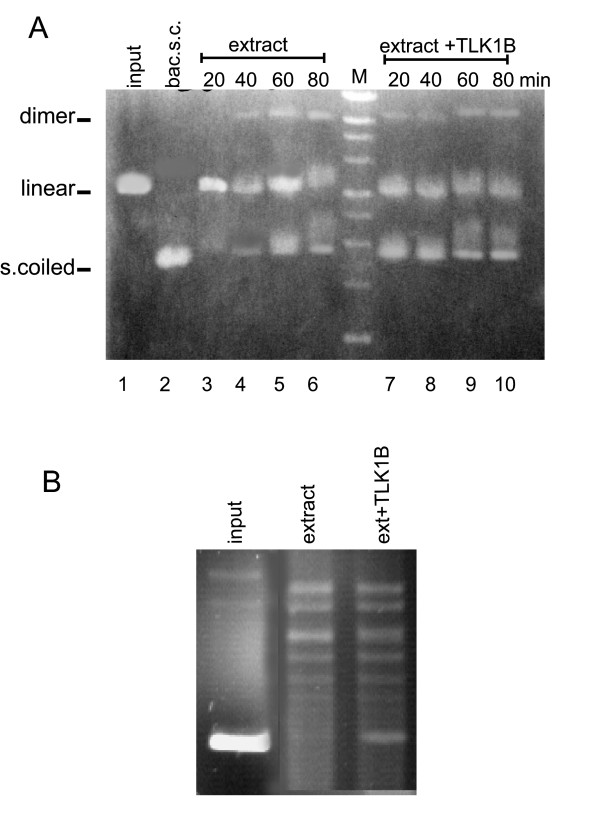
**A. Ligation reactions and supercoiling**. Reactions were prepared as described Experimental Procedures. The position of linearized plasmid (lane 1, input), bacterial supercoiled (lane 2) and dimers is indicated. The various topomeric forms of supercoiled plasmid (the result of deposition of nucleosomes on the template) are not resolved well in this gel without chloroquine, and appear as a slight smear (lanes 6, 9, 10). **B. Assay of chromatin assembly**. Nucloesomes assembly was carried out on 2 μg of Bluescript plasmid as described in Methods. Each band corresponds to the addition of one nucleosome, which decreases the linking number. These gels are representative of two different experiments.

The direct way to probe the function of TLK1B in repair of a DSB is *in vitro*. Repair assays were carried out as described in Methods, based on a system that demonstrated a synergism between CAF-1 and Asf1 in a repair-coupled nucleosome assembly [23]. If TLK1B increases the activity of Asf1 *in vitro*, then reactions supplemented with TLK1B should show a greater proportion of supercoiled topomers in addition to more ligated (closed) plasmid. Briefly, repair/nucleosome assembly was carried out on Bluescript plasmid that is linearized with EcoRI. We wished to monitor simultaneously: 1) Processing/ligation of the ends; and 2) superhelicity of the plasmid by the formation of nucleosomes on the template. Reactions contained nuclear extract, an energy mix, and additional purified TLK1B. The plasmid was then re-extracted and analyzed by electrophoresis in agarose gels. In Fig. 7A, linearized plasmids were incubated with cell extract +/- TLK1B, and repair was monitored during a time course. Note that the addition of TLK1B greatly stimulated the speed of the reaction in the formation of dimer molecules and the ligated/supercoiled forms of the plasmids, which already appear at 20 min instead of 40–60 min for extract without TLK1B. For shorter time points of incubation we used a more sensitive and quantitative method to monitor repair of the cut plasmids, *i.e*., a bacterial transformation assay. Linear plasmids do not transform bacteria, whereas repaired, covalently closed plasmids do. After 10 min of incubation with cell extract, in a typical experiment, we obtained 31 colonies vs. 260 for extract supplemented with exogenous TLK1B. Therefore, it appears that TLK1B improves the accessibility of the repair and ligation machinery to the free ends of the DNA or that it stimulates the deposition of histones [by Asf1; [11]] to assemble chromatin. This lends strong support to our hypothesis that the principal mechanism of increased radioresistance is through more efficient repair of DSBs likely linked to chromatin remodeling.

In Fig. 7B, we show an assay of supercoiling activity, which is a measure of chromatin assembly. In this assay, the plasmid Bluescript (nearly 100% supercoiled, *input*) is used as a template for the deposition of core histones in the presence of nuclear extract and an energy mix. In the absence of histones, the extract causes the bacterially supercoiled form to convert to mostly relaxed forms due to endogenous topoisomerases (data not shown). However, after incubation in the presence of histones, the plasmid migrates as a series of discrete supercoiled forms due to the formation of nucleosomes, which decrease the linking number by one integer per nucleosome. The addition of recombinant TLK1B stimulated the formation of the more highly supercoiled forms, particularly the form that runs like bacterially supercoiled plasmid.

## Discussion

In this work, we have provided four lines of evidence that the *Tousled *kinase, TLK1B, protects the cells from IR by facilitating the repair of DSBs. First, the pattern of phosphorylation/dephosphorylation of H2AX and H3 indicated that cells overexpressing TLK1B return to pre-IR phosphorylation state much more rapidly than controls. Second, the repair of episomes damaged with DSBs was much more rapid and complete by 8 hr of recovery in cells overexpressing TLK1B. Third, we have found that the repair of genomic breaks occurs more rapidly in cells overexpressing TLK1B, and with kinetics that are similar to those of repair of episomes. Lastly, we demonstrated with an *in vitro *repair system that the addition of recombinant TLK1B promotes repair of a linearized plasmid incubated with nuclear extract. Consistent with the results published by Groth [4] and Kodym [24] we found that TLK1 activity is inhibited by IR, as shown by loss of phosphorylation of histone H3, which is one of the best substrates of TLK1. Nonetheless, when TLK1B is overexpressed (about 6-fold in our stably transfected MM3MG cells), the recovery of H3 phosphorylation was quite rapid (about 4 hr) probably because of mass action due to higher levels of the kinase.

The role of TLK1B in radioresistance is particularly intriguing based on the recent findings that Asf1 is also a specific TLK1 substrate [11] known to participate with Rad53 in chromatin remodeling at sites of DSBs [25]. Furthermore, the importance of histone H3 kinases cannot be overstated. Phosphorylation of H3 at S^10 ^is becoming one of the most intensely studied aspects of chromatin remodeling, both during segregation of chromosomes at mitosis, and in aspects of transcription [13,26-28]. Therefore, studies of TLK1B and the family of *Tousled *kinases are bound to become the center of much attention. Elevated phosphorylation of H3 has also been reported in several lines of oncogenically transformed fibroblasts [29], although the underlying mechanism is unknown. We have found that elevated expression of TLK1B did not oncogenically transform MM3MG cells, but the cells became highly resistant to IR or doxorubicin [8]. We have preliminary results that demonstrate that TLK1B is elevated in some breast carcinomas and it is possible that this may result in a disease refractory to treatment [30]. We currently favor a mechanism by which TLK1B protects the cells from DSBs, by promoting repair-coupled chromatin remodeling which depends on the phosphorylation of histone H3 and Asf1.

Radiation-induced damage has been shown to result in rapid phosphorylation of histone H2AX. Phosphorylated foci of H2AX co-localize with DNA repair and signaling proteins, and H2AX has been demonstrated to be involved in their recruitment to sites of DSBs [31]. Knockout of H2AX in mice resulted in defects in concentration of repair proteins (53BP1, BRCA1, NBS1) at the sites of DNA damage, and H2AX-deficient cells are IR sensitive demonstrating the requirement of H2AX in DNA repair [15]. The importance of radiation-induced phosphorylation of H2AX in repair of DSBs has been shown by the fact that H2AX deficiency results in genomic instability [32]. In yeast, H2AX is not involved in activation of the S-phase checkpoint by DSBs, but rather in the efficiency of DNA repair [33]. The importance of histone H3 phosphorylation in DNA damage has not been investigated as well as that of H2AX, but the overall effect of these modifications is likely to be chromatin remodeling and recruitment of repair proteins. Recently, H2AX was found to recruit the chromatin remodeling protein INO80 [34].

Our current model is that in normal cells TLK1 (the constitutively expressed larger isoform) performs the normal functions of this kinase, which may have a role in chromatin remodeling and genome surveillance in unstressed conditions. Following DNA damage by IR or doxorubicin, synthesis of TLK1B is induced through a translational control mechanism [12]. TLK1B can then facilitate repair of DNA damage. This would greatly accelerate the response of those cells to DNA damage and the efficiency with which repair is implemented, significantly increasing their resistance to IR.

An alternative that we considered for a role of TLK1B in radioprotection is that TLK1B functions in a signaling pathway that protects cells from undergoing apoptosis. There are two compelling reasons why this is not likely (at least not directly). First, overexpression of TLK1B did not change the transcriptome in MM3MG cells (microarray analysis, data not shown), indicating that its protective effect is post-transcriptional. There were no changes in the expression of pro- and anti-apoptotic genes, or cell cycle regulators. Second, expression of TLK1B conferred protection against IR even in yeast. Whereas proteins involved in sensing and repairing DNA damage are conserved between mammals and yeast, prototypical proteins involved in the apoptotic and antiapoptotic pathways are not found in yeast.

## Conclusion

Studies of the *Tousled *kinases are only now beginning to shed light upon their function, despite the early discovery of a role in flower and leaf morphology inferred by mutations in plants. In this work, we have provided four lines of evidence that the *Tousled *kinase, TLK1B, protects the cells from IR by facilitating the repair of DSBs. First, the pattern of phosphorylation/dephosphorylation of H2AX and H3 indicated that cells overexpressing TLK1B return to pre-IR phosphorylation state much more rapidly than controls. Second, the repair of episomes damaged with DSBs was much more rapid and complete by 8 hr of recovery in cells overexpressing TLK1B. Third, we have found that the repair of genomic breaks occurs more rapidly in cells overexpressing TLK1B, and with kinetics that are similar to those of repair of episomes. Lastly, we demonstrated with an *in vitro *repair system that the addition of recombinant TLK1B promotes repair of a linearized plasmid incubated with nuclear extract. Therefore, it appears that TLK1 and TLK1B have a role in genome surveillance, particularly upon genotoxic stress, which induces the expression of TLK1B.

## Methods

### Cell lines and tissue culture

Normal breast epithelial cells, MM3MG, transfected or not with TLK1B were cultured as described in Li *et al*. [8].

### Radiation experiments

Control MM3MG cells and the cells overexpressing TLK1B [MM3MG-TLK1B; [8]] were harvested with PBS/EDTA and adjusted to 10,000 cells/tube in DMEM/10% FCS. Cells were irradiated in the Radiation department at LSUHSC with Elekta Precise linear accelerator at 6 MV. For each radiation dose levels (0 to 8 Gy), aliquots of serially diluted cells (100–5000) were plated on 6-well plates in triplicate. After a period of 10 days of incubation, the wells were rinsed with PBS and stained with crystal violet, and the colonies counted. The experiment was repeated thrice, and the results were expressed as the fraction of surviving cells compared to the number of colonies formed in the non-irradiated samples (plating efficiency).

### Western blots

The anti-histone H3 phosphorylated at Ser-10 and anti-histone H2AX phosphorylated at Ser-139 were from Upstate Cell Signaling (Lake Placid, NY). For Western blots, 30 μg of protein of each sample was separated on a 15% SDS/PAGE gel. The proteins were transferred to Immobilon-P membranes (Millipore, Bedford, MA) and incubated overnight with primary antiserum and for 1 hr with secondary antisera (1:1000 dil.). Finally, the membranes were washed three times and developed with Opti-4CN reagent (Bio-Rad, Hercules, CA).

### Extraction of episomes

Episomes were isolated from 2 × 10^7 ^cells (90% confluent flasks) stably transfected with empty vector (BK-Shuttle) or vector carrying TLK1B. Two methods were used to extract the episomes: either the standard Hirt's supernatant protocol [35], or alkaline lysis. Briefly, the cells were resuspended in 0.1 ml TE, lysed at room temperature with solution 1 (0.2 ml of 0.2 M NaOH, 1% SDS), which was then neutralized with solution 2 (0.15 ml of 3 M K-Acetate/glacial acetic acid). After a brief centrifugation at 10,000 × g to remove insoluble material (including genomic DNA), remaining nucleic acids were extracted with Phenol/Chloroform (1:1), and precipitated with cold EtOH. The episomes were analyzed by gel electrophoresis on 1% agarose/TAE, and stained with EtBr.

### Assay of genomic repair

To assess the repair of DNA damage *in vivo*, the modified TUNEL assay (terminal deoxynucleotidyltransferase-mediated dUTP nick end labeling) was applied. MM3MG or TLK1B cells were grown to 50% confluence on tissue culture slides prior to exposing them to ionizing irradiation (20 Gy). After radiation, the cells were allowed to recover for varying times (0, 2, 8 hr). Subsequently, cells were fixed in 4% formalin/PBS and permeabilized in 0.2% Triton-X100/PBS. For labeling DNA breaks *in situ*, the DeadEnd Colorimetric TUNEL System (Promega, Madison, WI) was used according to the manufacturer's protocol. The biotinylated nucleotides incorporated at 3'OH ends were reacted with horseradish peroxidase labeled-streptavidin, which was then detected by diaminobenzidine (DAB). The nuclear DNA-labeled sites (brown spots) within each cell were counted under a light microscope (40 × magnification), and the average (± SD) number of DNA breaks per cell was calculated. At least 10 cells per dose were counted.

### Assay of DSB repair *in vitro*

Nuclear extract from MM3MG cells was prepared as described in [36]. Repair assays were carried out as described by Mello [23]. Briefly, repair/nucloesome assembly was carried out on 0.1 μg of Bluescript plasmid (per reaction) that was linearized with EcoRI. We monitored simultaneously ligation of the ends and superhelicity of the plasmid by the formation of nucleosomes on the template. Reactions contain 20 μg of nuclear extract, 5 mM MgCl_2_, 40 mM Hepes, pH7.8, 0.5 mM DTT, 4 mM ATP, 20 μM dNTPs, 4 mM phosphocreatine, 2 u of creatine phosphokinase, and additional recombinant TLK1B. After incubation at 37°C for the indicated amount of time, the reactions were deproteinized with phenol and the plasmid was re-precipitated with cold EtOH.

### Assay of chromatin assembly

Nucloesomes assembly was carried out on 2 μg of Bluescript plasmid. Reactions contained 15 μg of MM3MG cell extract (which already contains sufficient amounts of topoisomerases), 5 mM MgCl2, 40 mM Hepes, pH 7.8, 0.5 mM DTT, 4 mM ATP, 20 μM dNTPs, 4 mM phosphocreatine, 2 u of creatine phosphokinase, and additional purified proteins (200 ng TLK1B and 2 μg supplemental HeLa histones). The reactions were incubated at 37°C for 0.5 hr. The plasmid was re- extracted with GeneClean III kit (Bio 101, Vista, CA), separated on an agarose gel and subsequently stained with EtBr.

## Authors' contributions

GSD prepared figures 1, 2, 3, 4. SKB prepared figure 7A; SS and SN prepared figure 5; SS prepared figure 6 and figure 7B. ADB wrote the paper.

## Figures and Tables

**Table 1:** 

RECOVERY TIME (hr)	MM3MG (number of breaks)	TLK1B (number of breaks)
0	13 +/- 0.35	11 +/- 2.83
2	11 +/- 0.64	5 +/- 1.06
8	7 +/- 1.91	4 +/- 1.27

* Non irradiated cells had no convincing labeled spots.
